# Hematology and Clinical Chemistry Reference Ranges for Laboratory-Bred Natal Multimammate Mice (*Mastomys natalensis*)

**DOI:** 10.3390/v13020187

**Published:** 2021-01-27

**Authors:** David M. Wozniak, Norman Kirchoff, Katharina Hansen-Kant, Nafomon Sogoba, David Safronetz, Joseph Prescott

**Affiliations:** 1 ZBS5—Biosafety Level-4 Laboratory, Robert Koch-Institute, 13353 Berlin, Germany; wozniakd@rki.de (D.M.W.); KirchoffN@rki.de (N.K.); Hansen-KantK@rki.de (K.H.-K.); 2 International Center for Excellence in Research, Malaria Research and Training Center, Faculty of Medicine, Pharmacy and Dentistry, University of Sciences, Techniques and Technologies of Bamako, Bamako 91094, Mali; nafomon@icermali.org; 3 Laboratory of Virology, Rocky Mountain Laboratories, National Institute of Allergy and Infectious Diseases, National Institutes of Health, Hamilton, MT 59840, USA; david.safronetz@canada.ca; 4 Zoonotic Diseases and Special Pathogens, National Microbiology Laboratory, Public Health Agency of Canada, Winnipeg, MB R3E 3M4, Canada

**Keywords:** *Mastomys natalensis*, multimammate mouse, multimammate rat, blood, reference value, normal value, baseline, clinical chemistry, Lassa virus

## Abstract

Laboratory-controlled physiological data for the multimammate rat (*Mastomys natalensis*) are scarce, despite this species being a known reservoir and vector for zoonotic viruses, including the highly pathogenic Lassa virus, as well as other arenaviruses and many species of bacteria. For this reason, *M. natalensis* is an important rodent for the study of host-virus interactions within laboratory settings. Herein, we provide basic blood parameters for age- and sex-distributed animals in regards to blood counts, cell phenotypes and serum chemistry of a specific-pathogen-monitored *M.* *natalensis* breeding colony, to facilitate scientific insight into this important and widespread rodent species.

## 1. Introduction

*Mastomys natalensis* are commensal wild-living rodents of the African continent with a large host range [1,2]. Even though *M. natalensis* are often described as “rats”, phylogenetically, they are more closely related to mice (*Mus*) than *Rattus* [3]. However, since they grow to sizes and weights greater than *Mus musculus* [4], the confusion with rats as a general descriptor is understandable. Seasonal resource limitations [5,6], their grani- and omnivore sustenance and the high reproduction capabilities of *M. natalensis* [5] cause large annual fluctuations of local populations. During the dry season, *M. natalensis* often live peri-domestically and are found inside human dwellings [6] consuming food scraps and unsecured food stores and thereby contaminate these with their excretions.

*Mastomys* sp. can harbor a diversity of zoonotic pathogens of serious concern for humans, including *Capillaria hepatica, Gongylonema* sp., *Escherichia coli*, *Salmonella typhimurium* [4,7], and, in the case of the visually indistinguishable sister species, *M. coucha, Yersinia pestis* infection, which could have been partially responsible for human plague clusters in Africa [8]. The pathogen of highest concern harbored by *M. natalensis*, as well as other species of Muroinae, including *Hylomyscus pamfi, Mus baoulei,* and *Mastomys erythroleucus,* is Lassa virus (LASV, *Lassa mammarenavirus, Arenaviridae*) [9,10,11], which is considered a Risk Group 4 (RG-4) pathogen. LASV is the etiological agent of the hemorrhagic virus disease called Lassa fever, which is considered a major public health concern in West African countries due to annual outbreaks and high morbidity and mortality.

*Mastomys* species, especially *Mastomys coucha*, have a long standing history as a model for schistosomiasis [12,13], the plague [14] and papilloma virus research [15]. However, basic blood parameters of *M. natalensis* from controlled rearing are not available. Additionally, up to this point and only recently, one annotated genome has been published of a laboratory strain of *M. coucha* [16,17]. Therefore, biomolecular analysis testing of *M. natalensis* is still based on genetic sequences acquired from *M. coucha*, which due to the close relationship have at least a chance to similarly work on *M. natalensis*.

To effectively study *M. natalensis* in laboratory settings, new tools and baselines parameters are currently being developed and established. Herein, we report the normal hematological and clinical chemistry values of healthy pathogen-tested animals of the animal species of *M. natalensis* from our laboratory-bred colony to further facilitate research on this currently understudied, yet important for human health, rodent species.

## 2. Materials and Methods 

### 2.1. Animals

The *Mastomys natalensis* colony originated from Mali and was brought to the USA in 2013 and has since bred successfully in captivity. Courtesy of Heinz Feldmann and David Safronetz, an off-shoot colony was established at the Bernhard Nocht-Institute for Tropical Medicine (BNITM) in Hamburg, Germany, in 2017, with another off-shoot colony being established at the Robert Koch-Institute (RKI) in Berlin, descending from the BNITM colony in 2018.

The animals were bred at the Animal Husbandry Facility of the Robert Koch-Institute in (RKI) Berlin. Groups of 2–4 animals of the same sex were co-housed in filter-top cages (2000P Tecniplast) with small wood chip bedding and paper nesting material at 22 °C, 40–50% relative humidity on standard rodent chow and water ad libitum during a 12-h light cycle. The animals used for the study were between 8 and 53 weeks of age (specifically 8, 12, 17, 24, 27, 51, 52, and 56 weeks). The calculated reference ranges are deduced from not completely sex-matched groups (40.7% male against 59.3% females) before removal of statistical outliers. The specific age stratification can be seen in Appendix A Table A1.

According to analyses on sentinel animals, the colony was free of most specific pathogens tested with the exception of *Rodentibacter* sp. (serological), *Helicobacter* spp. (PCR) and *Chilomastix* spp. (microscopic). The full list of tested organisms can be seen in Appendix A Table A2.

### 2.2. Blood Collection

*M. natalensis* were anesthetized with an isoflurane overdose and exsanguinated via cardiac puncture using BD vacutainer system (K2 EDTA 2.0 mL, SST-II 2.5 mL; 21G or 22G needle adapter). The euthanasia conformed to German animal protection laws and was registered under animal euthanasia report number T0168-19 with the Landesamt für Gesundheit und Soziales in Berlin, Germany.

### 2.3. Blood Cell Counts

Whole blood that was collected in tubes containing EDTA (BD Vacutainer K2EDTA) was agitated on a rotator at 6 rpm until measured with an automatic hematology analyzer (Abaxis HM5c). The hematology analyzer was maintained according to manufacturer instructions and correct performance was verified using commercial quality controls of normal and high-range reference blood (Abaxis VetScan HM5 Hematology Control). The samples were measured using the standard mouse settings, without modifications. In addition to automatic hematological analysis, thin-film blood smears stained with Diff-Quik staining (Labor + Technik: LT-SYS^®^) were prepared from the identical samples, and used for microscopic visualization and manual counting verification. Micrographs were captured using analysis 5.0 (Olympus) software and panels were composed using paint.net v4.1 (dotPDN LLC).

### 2.4. Clinical Serum Chemistry

Blood that was collected in serum separation tubes (BD Vacutainer SST II) was left to clot for at least 30 min and subsequently centrifuged at 3000× *g* for 5 min. The sera were aliquoted and stored at −80 °C until measured on an automatic clinical chemistry analyzer (FUJI DRI-CHEM NX500i) with the corresponding colorimetric test assays (FUJI). The samples were assessed for concentrations of creatinine (CRE), blood urea nitrogen (BUN), albumin (ALB), Glutamic pyruvate transaminase/Alanine transaminase (GPT/ALT), glutamic oxaloacetic transaminase/Aspartate transaminase (GOT/AST) and Alkaline phosphatase (ALP). If required, due to volume constraints, the samples were diluted with sterile 0.9% NaCl solution.

### 2.5. Data Analysis and Statistics

Data were analyzed using Graphpad Prism 8.4. Strongly hemolytic sera were removed from analysis prior to identification of statistical outliers. Statistical outliers for each group were identified using Robust Regression and Outlier removal (ROUT method) with a Q-coefficient of 1%. Measurements below the limit of detection in an assay were imputed by using $\frac{l.o.d.}{\sqrt{2}}$ as set value. One animal was removed from the analysis due to a skin condition that appeared inflammatory. Normal reference ranges were established by calculating the 2.5% and 97.5% percentiles (identical to total range when *n* < 40). Multiple *t*-tests were performed on normally-distributed parametric data using Holm–Sidak correction when not stated differently. Non-parametric data was compared using Mann–Whitney rank tests and *p*-values adjusted according to Sidak correction.

## 3. Results

### 3.1. Blood Cell Phenotypes in Thin-Film Blood Smears

**Figure 1:**
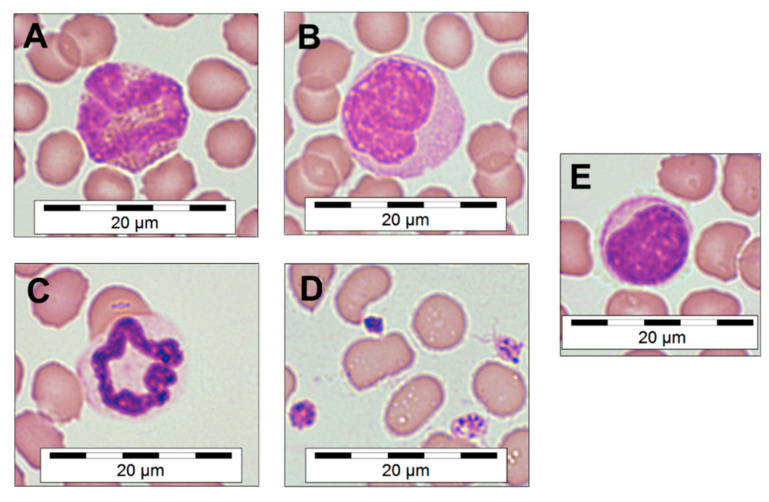
Microscopic morphology of *M. natalensis* blood cells. Eosinophil (**A**), monocyte (**B**), neutrophil (**C**), activated platelets (**D**) and lymphocyte (**E**). Quick-Diff staining, magnification 100× (oil immersion). Microscopic photographs were digitally sharpened and saturation increased by 8% and a scale bar was overlaid.

Visual characterization of blood cells from dried EDTA thin films allowed for categorization of the common blood cell types typically found in *Mammalia* and to measure their diameters (Table 1). During the visual screening of the blood samples, no basophils could be identified. The modified Romanowsky-stained thin-film blood smears (Figure 1) also provided information about the phenotype of the red blood cells, which sometimes presented with a spiked phenotype, likely because of a possible overconcentration of EDTA within the incompletely filled commercial tubes. Additionally, activated platelets were observed in the majority of samples, identified by their early dendritic and pseudopodia-rich phenotype (Figure 1D).

### 3.2. Blood Cell Reference Ranges

**Figure 2:**
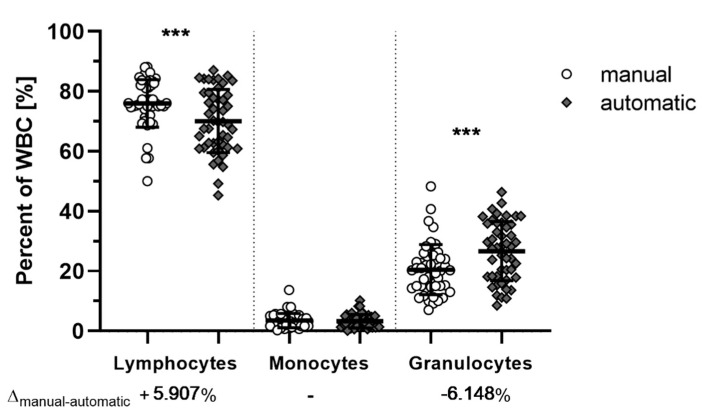
Manual and automatic white blood cell counts of *M. natalensis*. Both manual (white) and automatic (grey) counts of ETDA blood achieved similar results. Measurements between manual and automatic enumeration of lymphocytes (*p =* 0.0009) and granulocytes (*p =* 0.0003) are significantly different with differences of the mean (Δ_manual-automatic_) at around 6% (95% CI: 2.2 to 9.6 (lymphocytes) or −9.7 to −2.6 (neutrophils). Data shown as biological replicates (symbol) and mean (bar) ± SD. Mixed-model fitted multiple comparison paired *t*-test with Bonferroni adjusted *p*-values. ***: Significant differences (*p* < 0.001).

Since *M. natalensis* in not a classically-researched species in veterinary sciences or laboratories, commercial hematological offerings are not validated for this species. Manual counting of stained thin blood smears provided a general consensus of cell type ratios to compare with the hematology analyzer using the mouse setting (Figure 2). While significant differences between manual and automated counting of lymphocytes (*p* = 0.0009) and granulocytes (*p =* 0.0003) were observed, the inversely proportional mean differences of about +6% and −6% in the manual count of lymphocytes and granulocytes, respectively, indicate that this difference is likely a stable shift due to miscategorization of lymphocytes as granulocytes by the automated hematology analyzer in the mouse setting. Nevertheless, both methods were able to identify abnormal blood samples.

Using automated hematology analysis, white blood cell (WBC) concentrations were determined and identified as lymphocytes (LYM), monocytes (MON), or granulocytes (GRA) (Table 2). On the basis of these values, reference ranges were established after the ROUT method outlier removal.

**Figure 3:**
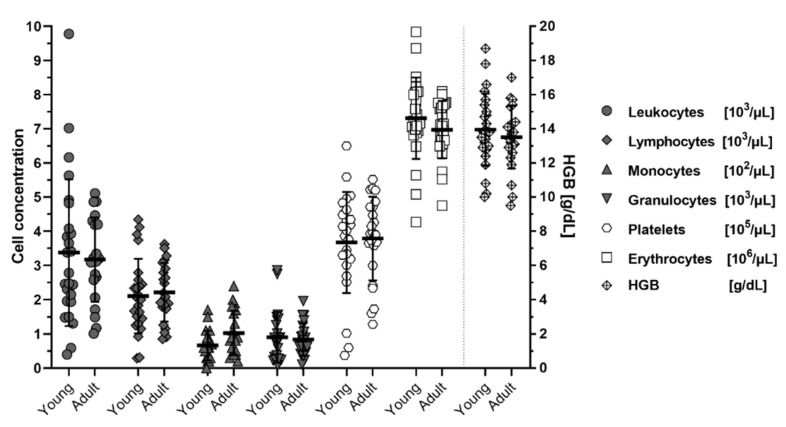
Cell concentrations in blood of *M. natalensis* with age. Young (<6 months; 8–17 weeks) and adult (>6 months; 24–56 weeks old) animals show no significant differences in blood cell counts or hemoglobin concentrations (HGB). Data shown as biological replicates (symbol), mean (bar) ± SD.

When analyzed for age-effects on blood cell concentrations, no differences could be discerned between animals younger than 6 months (<24 weeks) of age and older than 6 months (>24 weeks) except for a minor non-significant trend (*p =* 0.208 Dunnet T3 corrected; *p =* 0.040 Welch’s t-test, uncorrected) of increased monocytes in older individuals (Figure 3). Age and sex distribution of the individuals can be seen in Appendix A table (Table A1).

Automated determination of red blood cell concentrations (RBC), hematocrit value (HCT) and hemoglobin concentration (HGB) and platelet concentrations (PLT) in the blood revealed no sex-specific differences in *M. natalensis* (Table 3). Female *M. natalensis* overall displayed higher standard deviation of these red blood parameters.

### 3.3. Serum Reference Ranges

The sera from cardiac punctures were analyzed for six parameters (Table 4); CRE, BUN, ALB, GPT/ALT, GOT/AST, and ALP. Sex-specific differences were observed for ALB, BUN, and AST, with females displaying higher ALB (*p =* 0.013) and AST (*p =* 0.007) concentrations compared to males, while BUN (*p =* 0.004) was significantly lower.

The observed differences in CRE levels have to be loosely interpreted because 44.8% of the CRE data set was imputed due to measurements below the level of detection (LOD = 0.2 mg/dL). Despite females being overrepresented in the imputed data set, binominal testing of each sex’s ratio of animals below LOD against the ratio of the other group (male observed vs. female expectation percentages: *p =* 0.161; female observed vs. male expectation percentages: *p =* 0.071), or a 50% expectation (male *p =* 0.169; female *p* > 0.999), showed that this result is likely still a random effect due to a low number of animals tested.

## 4. Discussion

*Mastomys natalensis* is an understudied rodent species with importance as a reservoir and vector for many human pathogens, including the highly pathogenic Lassa virus, for which almost no specific analytical tools have been established. Herein, we present hematological data showing that blood cell counts of young animals do not differ from older animals, as well clinical-chemistry data showing few but significant differences between males and females for this laboratory-bred wild animal species.

In this first effort to study *M. natalensis* under laboratory conditions, we were able to generate reference ranges for white blood cells, lymphocytes, monocytes, granulocytes, red blood cells, and platelets in the blood and specify their phenotypical appearance and sizes.

The final cell counts in this study were obtained by use of Coulter-principle-based automatic counting. Since the analysis panel of the employed automatic hematology analyzer are not optimized for *M. natalensis* samples, lymphocyte concentrations were generally understated, while granulocytes concentrations were overstated by the automatic counter when compared to manual differential counting. This could be caused by insufficient lymphocyte lysis of the *M. natalensis* blood when using the standard mouse settings of the machine. This counting discrepancy might be solvable by increasing the lysis buffer volume for better automatic differentiation, but has to be tested in the future.

Our determined hematological reference ranges are comparable to a smaller and more limited previously published study that used inbred *M. natalensis* [18] despite differing blood sampling techniques employed in the studies, which generally can affect the cell composition of the blood [19,20]. Similarly, hematological data of inbred *M. coucha* is consistent with our data [21], even though the published data on *M. coucha* shows lower standard deviations than the data of *M. natalensis* in this report. Publications prior to the year 1980, or other publications on the “GRA Giessen-strain” or “*Praomys (Mastomys) natalensis*” [22], have to be considered carefully. The species name *M. natalensis* has previously been assigned to a complex of several externally identical sympatrically-living *Mastomys* species. Karyotyping analyses later identified the “GRA Giessen-strain” of “*M. natalensis*” as the species *M. coucha* [23,24] under inbred characteristics.

While basophilic granulocytes are often studied in *M. coucha* during filarial infections [25,26], our study of healthy helminth-free *M. natalensis* did not allow us to document the characteristics of this rarer cell population. Comparing *M. natalensis* to other typical laboratory rodents, the measured leukocyte concentrations are much closer to reference ranges of the highly standardized C57BL/6 mouse strain, matching their closer genetic relation to mice [3], than to those of rats.

To date, no clinical chemistry reference ranges for *M. natalensis* are available in the literature. Literature stating clinical-chemistry parameters for *M. natalensis,* especially in the research of anti-filarial and anti-plasmodialdrugs, are historically again limited on the Giessen-strain of *Mastomys coucha* [27]. We therefore provide reference ranges for creatinine, blood urea nitrogen, albumin, alanine-amino-transferase, aspartate-amino-transferase, and alkaline phosphatase true to *M. natalensis* and could observe sex-dependent difference in BUN, ALB, and AST concentrations. Comparison of published *M. coucha* parameters shows similar mean concentrations of GPT/ALT with 21.6 ± 2.2 U/L to our 21.2 ± 7.1 U/L, while GOT/AST values in the *M. coucha* Giessen-strain generally ranged lower (40.2 ± 3.2 U/L vs. 95.2 ± 30.7 U/L), which might be attributed to different sampling techniques or the maintained genetic heterogeneity of the *M. natalensis* colony used in this study.

Serum ALP concentrations of young *M. natalensis* was generally elevated (data not shown). As it is known that mammals in stages of bone growth show elevated levels of ALP in the serum, other liver-specific serum parameters, such as γ-glutamyl transferase (GGT), might need to be considered and established to accurately judge alterations in the liver function of young *M. natalensis*.

During analysis, several samples that had increased AST and ALT values, were excluded due to outlier testing. This could indicate that the cardiac sampling technique employed, which proved more difficult due to enhanced clotting capacities compared to *Mus musculus*, might have resulted sometimes in sub-optimal sample quality for these analytes. Additionally, the interindividual variation in our breeding colony could have affected the spread of the data. This has to be explored in the future.

In the literature, minor effects of isoflurane on white blood cell counts have been documented in mice [28]. This implies that the reference ranges reported herein should mainly be applied to animals euthanized in a similar fashion, and for blood collections via cardiac puncture [20].

Overall, this study showed that the reference ranges for *M. natalensis* clinical chemistry values fall between those of mice and rats [29,30,31] and are partially unique to the species, even when compared to *M. coucha*.

Past routine serological and PCR analysis of sentinel animals from our colony indicated a presence of *Mastophorus muris.* However, the fact that no basophils were found, might indicate that this infection in the colony might be cleared or was restricted to very few animals. Few specific pathogens were also found in the studied *M. natalensis* colony during routine testing (Table A2). Thus, the parameter ranges presented here might not completely apply to animals of higher sanitisation standards. While all animals analyzed in this study presented as healthy, the effects of these commensal microorganisms on our results cannot be assesed at this time point, as also no metagenomic data on any *M. natalensis* breeding colony is available. Other non-specifically tested pathogens, such as endogenous viruses or commensal microorganisms, could be part of the biota of these animals, which might further influence parameters measured in this study. Therefore, different reference ranges might need to be applied to different breeding colonies, also based on their different genetic heterogeneity or geographical origin. Hence, results of this study should not be applied for veterinary diagnostic purposes of *M. natalensis* of other origins.

Taken together, no specific WBC-count differences could be observed between *M. natalensis* younger than six months and older than six months (up to one year of age), even though important immunological differences could still exist. Valuable insights were gathered by identifying differing clinical-chemistry reference ranges for female and male individuals for BUN, AST/GOT, and ALT/GPT, which can improve future experimental design with these animals, especially against the background of their role as reservoir hosts of LASV.

## Figures and Tables

**Table 1:** Blood cell sizes of *Mastomys natalensis*. Cardiac blood smear slides stained with Diff-Quik staining were analyzed microscopically, magnification 100× (oil immersion).

Cell Type	Mean Size (±SD)	Median Size
Erythrocyte (*n* = 47)	6.1 ± 0.4 µm	6.1 µm
Thrombocyte (*n* = 28)	2.2 ± 0.6 µm	2.1 µm
Monocyte (*n* = 10)	14.6 ± 2.0 µm	14.4 µm
Lymphocyte (*n* = 13)	9.1 ± 1.3 µm	8.8 µm
Neutrophil (*n* = 14)	13.1 ± 1.1 µm	13.1 µm
Eosinophil (*n* = 11)	12.4 ± 1.4 µm	12.7 µm

**Table 2:** White blood cell reference ranges of *M. natalensis*. Blood cell concentrations from cardiac puncture according to automated Coulter-counting and additional lymphocyte and granulocyte correction according to manual count verification of the samples. Standard deviation (σ)**.**

	Mean (Reference Range)	σ	Median	Mean and Reference Range with 6%-Correction of Automatic Counts (see Figure 2)
WBC [10³/µL] (*n* = 45)	3.138 (0.43–6.89)	1.491	3.12	-
LYM [10³/µL] (*n* = 47)	2.239 (0.29–5.64)	1.114	2.09	2.373 (0.31–5.98)
MON [10³/µL] (*n* = 42)	0.084 (0.00–0.06)	0.057	0.07	-
GRA [10³/µL] (*n* = 43)	0.783 (0.08–1.92)	0.477	0.79	0.736 (0.08–1.80)

**Table 3:** Red blood cell hematologic parameters of male and female *M. natalensis.* The 2.5–97.5%-percentile is identical to the overall range since *n* < 40. Paired *t*-test with Holm-Sidak correction.

Males ♂	Mean (*Reference Range*)	σ	Median	Holm–Sidak Adjusted *p*-Values between Sexes	Females ♀	Mean (*Reference Range*)	σ	Median
RBC [10^6^/µL] (*n* = 22)	7.524 (*5.64–8.52*)	0.659	7.650	0.12	RBC [10^6^/µL] (*n* = 25)	6.898 (*4.27–9.84*)	1.170	6.940
HCT [%] (*n* = 23)	43.75 (*32.06–50.02*)	4.45	43.83	0.44	HCT [%] (*n* = 24)	41.61 (*29.32–55.43*)	6.08	41.48
HGB [g/dL] (*n* = 23)	14.15 (*10–17*)	1.77	14.10	0.44	HGB [g/dL] (*n* = 24)	13.39 (*9.5–18.7*)	2.15	13.30
PLT [10³/µL] (*n* = 22)	363.5 (*60–522*)	116.0	374.5	0.66	PLT [10³/µL] (*n* = 25)	381.3 (*37–650*)	151.0	402.0

**Table 4:** Serum parameters of male and female *M. natalensis.*

Males ♂	Mean (*Reference Range*)	σ	Median	Sidak-Adjusted *p*-Values Mann-Whitney Test	Females ♀	Mean (*Reference Range*)	σ	Median
CRE [mg/dL] (*n =* 24)	0.271 (*0.14–0.58*)	0.117	0.26	<0.0006 *^a^*	Cre [mg/dL] (*n =* 24)	0.172 (*0.14–0.27*)	0.044	0.14
BUN [mg/dL] (*n =* 24)	26.18 (*16.0–42.6*)	7.78	24.1	0.0035 *	BUN [mg/dL] (*n =* 30)	19.08 (*3.5–28.1*)	4.99	18.5
ALB [g/dL] (*n =* 22)	2.56 (*1.4–4.0*)	0.64	2.6	0.0129 *	ALB [g/dL] (*n =* 30)	3.25 (*1.8–5.4*)	0.86	3.3
ALT/GPT [U/L] (*n =* 20)	21.2 (*12–38*)	7.1	20	>0.9999	ALT/GPT [U/L] (*n =* 22)	25.3 (*13–50*)	10.7	21.5
AST/GOT [U/L] (*n =* 20)	95.2 (*54–183*)	30.7	83	0.0065 *	AST/GOT [U/L] (*n =* 22)	138.2 (*80–296*)	54.9	120.5
ALP [U/L] (*n =* 22)	103.9 (*38–268*)	59.1	89	>0.9999	ALP [U/L] (*n =* 29)	111.3 (*10–247*)	67.9	88

*^a^*: Cre: ~45% of data imputed via $\frac{l.o.d.}{\sqrt{2}}$, statistical significance hence disputable. *: Significant differences (adjusted *p*-value < 0.05) between males and females in BUN, ALB, and GOT.

## Appendix A

**Table A1:** Age and sex distribution of analyzed *M. natalensis* prior to removal of outliers.

	Number		
Age (Weeks)	♂	♀	
8	5	6	<24 weeks: *n* = 29
12	2	3	
17	7 (9 *)	6 (12 *)	
24	2	0	>24 weeks: *n* = 22
27	3	7	
51	1	0	
52	3	2	
56	0	4	
	23 (25 *)	28 (34 *)	

* number of animals for serum analysis.

**Table A2:** Specific pathogen testing of sentinel animals from the *Mastomys natalensis* breeding colony.

Pathogen	Detection Method	Status
*Clostridium piliforme*	Serology	negative
*Rodentibacter* sp.	Serology	positive
*Streptobacillus moniformis*	Serology	negative
*Mastophorus muris*	Serology	Previously positive, now negative
*Mycoplasma pulmonis*	Serology	negative
Mouse Adenovirus FL	Serology	negative
Ectromelia virus	Serology	negative
Mouse Hepatitis Virus	Serology	negative
Murine K-Virus Polyoma virus	Serology	negative
Lymphocytic choriomeningitis virus	Serology	negative
Mouse Minute Virus	Serology	negative
Pneumonia virus of mice	Serology	negative
Polyoma-Virus	Serology	negative
Reovirus 3	Serology	negative
Sendai virus	Serology	negative
Theiler’s Murine Encephalomyelitis Virus GD VII	Serology	negative
Murine Rotavirus EDIM	Serology	negative
Lactate dehydrogenase elevating virus	Serology	negative
Hantaan Virus	Serology	negative
Mouse Adenovirus K87	Serology	negative
Murine cytomegalovirus	Serology	negative
Parvoviruses	Serology	negative
Murine norovirus 1	Serology	negative
Encephalomyocarditis virus	Serology	negative
Puumala Virus	Serology	negative
Mouse astrovirus	Serology	positive
Mastomys Natalensis Papilloma Virus	Serology	negative
*Citrobacter rodentium*	PCR/MALDITOF	negative
*Clostridium piliforme*	PCR/MALDITOF	negative
*Corynebacterium kutscheri*	PCR/MALDITOF	negative
*Escherichia coli*	PCR/MALDITOF	Previously positive, now negative
*Rodentibacter* spp.	PCR/MALDITOF	positive
*Salmonella* spp.	PCR/MALDITOF	negative
*Streptococcus ß-hemolytic*	PCR/MALDITOF	negative
*Streptococcus pneumoniae*	PCR/MALDITOF	negative
*Helicobacter* spp.	PCR/MALDITOF	positive
*Streptobacillus moniformis*	PCR/MALDITOF	negative
*Klebsiella oxytoca*	PCR/MALDITOF	negative
*Proteus mirablilis*	PCR/MALDITOF	Previously positive, now negative
*Pseudomonas aeruginosa*	PCR/MALDITOF	negative
*Staphylococcus aureus*	PCR/MALDITOF	negative
*Pneumocytis murina*	PCR/MALDITOF	negative
**ecto-arthropoda**	Microscopy	negative
*Aspiculuris* spp.	Microscopy	negative
*Syphacia* spp.	Microscopy	negative
*Chilomastix* spp.	Microscopy	positive
*Coccidia* spp.	Microscopy	negative
*Entamoeba* spp.	Microscopy	negative
*Giardia* spp.	Microscopy	negative
*Spironucleus muris*	Microscopy	negative
*Tritrichmonas* spp.	Microscopy	negative
**other flagellates**	Microscopy	negative

## References

[1] De Bellocq J.G., Bryjová A., Martynov A.A., Lavrenchenko L.A. (2020). Dhati Welel virus, the missing mammarenavirus of the widespread Mastomys natalensis. J. Vertebr. Biol..

[2] Mari Saez A., Cherif Haidara M., Camara A., Kourouma F., Sage M., Magassouba N., Fichet-Calvet E. (2018). Rodent control to fight Lassa fever: Evaluation and lessons learned from a 4-year study in Upper Guinea. PLoS Negl. Trop. Dis..

[3] Lecompte E., Aplin K., Denys C., Catzeflis F., Chades M., Chevret P. (2008). Phylogeny and biogeography of African Murinae based on mitochondrial and nuclear gene sequences, with a new tribal classification of the subfamily. BMC Evol. Biol..

[4] Demartini J.C., Green D.E., Monath T.P. (1975). Lassa virus infection in Mastomys natalensis in Sierra Leone. Gross and microscopic findings in infected and uninfected animals. Bull. World Health Organ..

[5] Leirs H., Verhagen R., Verheyen W. (1994). The Basis of Reproductive Seasonality in Mastomys Rats (Rodentia, Muridae) in Tanzania. J. Trop. Ecol..

[6] Fichet-Calvet E., Lecompte E., Koivogui L., Soropogui B., Doré A., Kourouma F., Sylla O., Daffis S., Koulémou K., Meulen J. (2007). Ter Fluctuation of Abundance and Lassa Virus Prevalence in Mastomys natalensis in Guinea, West Africa. Vector-Borne Zoonotic Dis..

[7] Fiedler L.A. (1994). Rodent Pest Management in Eastern Africa.

[8] Isaäcson M., Taylor P., Arntzen L. (1983). Ecology of plague in Africa: Response of indigenous wild rodents to experimental plague infection. Bull. World Health Organ..

[9] Olayemi A., Cadar D., Magassouba N., Obadare A., Kourouma F., Oyeyiola A., Fasogbon S., Igbokwe J., Rieger T., Bockholt S. (2016). New Hosts of The Lassa Virus. Sci. Rep..

[10] Yadouleton A., Agolinou A., Kourouma F., Saizonou R., Pahlmann M., Bedié S.K., Bankolé H., Becker-Ziaja B., Gbaguidi F., Thielebein A. (2019). Lassa virus in pygmy mice, Benin, 2016–2017. Emerg. Infect. Dis..

[11] Monath T.P., Newhouse V.F., Kemp G.E., Setzer H.W., Cacciapuoti A. (1974). Lassa virus isolation from Mastomys natalensis rodents during an epidemic in Sierra Leone. Science.

[12] Pitchford R.J., Visser P.S. (1962). The role of naturally infected wild rodents in the epidemiology of schistosomiasis in the Eastern Transvaal. Trans. R. Soc. Trop. Med. Hyg..

[13] Lämmler G., Petrányi G. (1971). Chemotherapeutic studies on experimental Schistosoma mansoni infection of Mastomys natalensis. Bull. World Health Organ..

[14] Arntzen L., Wadee A.A., Isaäcson M. (1991). Immune responses of two Mastomys sibling species to Yersinia pestis. Infect. Immun..

[15] Hasche D., Rösl F. (2019). Mastomys Species as Model Systems for Infectious Diseases. Viruses.

[16] Modlin I.M., Zucker K.A., Zdon M.J., Sussman J., Adrian T.E. (1988). Characteristics of the spontaneous gastric endocrine tumor of mastomys. J. Surg. Res..

[17] Hardin A., Nevonen K.A., Eckalbar W.L., Carbone L., Ahituv N. (2019). Comparative Genomic Characterization of the Multimammate Mouse Mastomys coucha. Mol. Biol. Evol..

[18] Kagira J.M., Maina N.W., Thuita J.K., Ngotho M., Hau J. (2005). Influence of cyclophosphamide on the haematological profile of laboratory bred African soft-furred rats (Mastomys natalensis). Scand. J. Lab. Anim. Sci..

[19] Doeing D.C., Borowicz J.L., Crockett E.T. (2003). Gender dimorphism in differential peripheral blood leukocyte counts in mice using cardiac, tail, foot, and saphenous vein puncture methods. BMC Clin. Pathol..

[20] Nemzek J.A., Bolgos G.L., Williams B.A., Remick D.G. (2001). Differences in normal values for murine white blood cell counts and other hematological parameters based on sampling site. Inflamm. Res..

[21] Ziegler C., Käufer-Weiss I., Zahner H. (1991). On the Pathogenesis of Anaemia and Leukopenia in Filarial (Litomosoides carinii) Infection of Mastomys natalensis. J. Vet. Med. Ser. B.

[22] Green C.A., Keogh H., Gordon D.H., Pinto M., Hartwig E.K. (1980). The distribution, identification, and naming of the Mastomys natalensis species complex in southern Africa (Rodentia: Muridae). J. Zool..

[23] Lecompte E., Brouat C., Duplantier J.M., Galan M., Granjon L., Loiseau A., Mouline K., Cosson J.F. (2005). Molecular identification of four cryptic species of Mastomys (Rodentia, Murinae). Biochem. Syst. Ecol..

[24] Duplantier J.M., Britton-Davidian J., Granjon L. (1990). Chromosomal characterization of three species of the genus Mastomys in Senegal. J. Zool. Syst. Evol. Res..

[25] Torrero M.N., Morris C.P., Mitre B.K., Hübner M.P., Fox E.M., Karasuyama H., Mitre E. (2013). Basophils help establish protective immunity induced by irradiated larval vaccination for filariasis. Vaccine.

[26] Joseph S.K., Verma S.K., Verma R., Saxena J.K., Srivastava M., Murthy P.K. (2013). Anti-inflammatory BmAFI of Brugia malayi modulates IgE, histamine and histamine receptor responses in Mastomys coucha. Acta Trop..

[27] Chander R., Srivastava V., Tandon And J.S., Kapoor N.K. (1995). Antihepatotoxic Activity of Diterpenes of Andrographis Paniculata (Kal-Megh) Against Plasmodium Berghei-Induced Hepatic Damage in Mastomys Natalensis. Int. J. Pharmacogn..

[28] Hoggatt J., Hoggatt A.F., Tate T.A., Fortman J., Pelus L.M. (2016). Bleeding the laboratory mouse: Not all methods are equal. Exp. Hematol..

[29] Charles River Lab C57BL/6 Mouse Hematology C57BL/6 Mouse Biochemistry North American Colonies—January 2008–December 2012. https://www.criver.com/sites/default/files/resources/C57BL6MouseModelInformationSheet.pdf.

[30] Giknis M.L.A., Clifford C. (2008). Clinical Laboratory Parameters for Crl: WI (Han).

[31] Jackson Laboratory Physiological Data Summary—C57BL/6J (000664). http://jackson.jax.org/rs/444-BUH-304/images/physiological_data_000664.pdf.

